# Toxicity of ZnFe-SO_4_ layered double hydroxide in *Tetradesmus obliquus* and evaluation of some physiological responses of the microalgae for stress management

**DOI:** 10.1038/s41598-023-51042-y

**Published:** 2024-01-10

**Authors:** Samaneh Torbati, Parisa Yekan Motlagh, Alireza Khataee

**Affiliations:** 1 Department of Ecology and Aquatic Stocks Management, Artemia and Aquacultur Research Institute, Urmia University, Urmia, 5756151818 Iran; 2 Research Laboratory of Advanced Water and Wastewater Treatment Processes, Department of Applied Chemistry, Faculty of Chemistry, University of Tabriz, Tabriz, 5166616471 Iran; 3 Department of Chemical Engineering, Istanbul Technical University, 34469 Maslak, Istanbul, Turkey

**Keywords:** Plant sciences, Environmental sciences, Nanoscience and technology

## Abstract

Layered double hydroxides (LDHs), regarding their physical and structural properties, have different and wide applications industry and their increasing use may raise ecological and human health concerns. However, the potential toxicity mechanisms of LDHs in different organisms are still unclear. In the present work, after synthesizing of ZnFe-SO_4_ LDH and studying of its characterization by XRD, FT-IR, SEM, EDX-mapping, TEM and Raman, its toxicity in *Tetradesmus obliquus* was evaluated. According to experimental results, the growth of the algae and content of photosynthetic pigments were significantly decreased after treatment with 100 mg/L of ZnFe-SO_4_ LDH. The high dose exposure to the LDH also inhibited the activity of SOD and POD enzymes, possibly due to the LDH- catalyzed reactive oxygen species production. In addition, lipid peroxidation and the content of phenolic compounds, as no-enzymatic antioxidants were increased by enhancement of the LDH concentration. The rise of phenol, flavonoids and MDA contents could be regarded as some manifestations and responses to the toxic effects of the contaminant in the algae cells. The results provided a better understanding of the undesirable effects and toxicity of LDHs in aquatic organisms.

## Introduction

During the last decade, layered double hydroxides (LDHs) nanomaterials have attracted noteworthy attention because of their outstanding features, including high specific surface area, anion exchange capability, catalytic ability, thermal stability, etc.^1^. These materials have different uses in various fields, such as biology, electrochemistry, pharmacology, and the removal of environmental pollutants^1,2^. Due to their low cost, chemical stability, high catalytic activity and absorbing nature LDHs have proven to be suitable materials for environmental remediation applications^3^. For instance, their high adsorption capacity for metal(loid)s, including As, as well as Cd, Cu, Pb and Zn, was confirmed^4^. They also exhibited proper execution for the degradation of some organic dyes and polycyclic aromatic hydrocarbons (PAHs)^5,6^. Furthermore, successful removal of ibuprofen, acetaminophen, diclofenac and arsenic by Zn-Fe mixed metal oxides was confirmed^7^.

Despite the unique applications of different nanomaterials, their hazardous effects and the associated mechanisms have also been widely studied in various organisms^8^. Direct interaction of nanomaterials with cell surface, dissolution of toxic elements and production of reactive oxygen species (ROS) were reported as three main events causing oxidative stress in biological systems^9^. However, there are a handful of studies have focused on the toxicity of LDHs in living organisms. Toxicity of a LDH on *Vibrio fischeri*, *Daphnia magna* and *Spirodela polyrhiza* was determined and *S. polyrhiza* was found to be the least sensitive organism to the LDH (EC_50_ = 800 mg/L)^3^. In other study, the growth of *Scenedesmus quadricauda* as a fresh water green algae was inhibited by 10 mg/L LDH (EC_50_ = 10 mg/L)^10^. Because of having simple growth conditions, short life cycle and sensitivity to different classes of pollutants, microalgae such as species of Scenedesmacease family are critical biomarkers for environmental changes and have been of much interest^11^. *Tetradesmus (Scenedesmus) obliquus* (Turpin) M.J. Wynne is a member of the family that has gained attention for the production of biocomponents that were used in wastewater treatment and for other environmental purposes^12^. The influential role of the microalgae has been well confirmed in the remediation of phosphorus, sulfate, phosphate, ammonium, phenolic compounds, heavy metals and some medicinal compounds such as paracetamol and salicylic acid^13–15^. The microalgae was also used for biofuel production, aquaculture, development of biosensors and applied as bioreactor like some cyanobacteria^16–18^. *T. obliquus* showed excellent sensitivity to pesticides, heavy metals and some toxic substances like formaldehyde and can be used for detecting pollutants in aqueous media^11^. However, despite the mentioned extensive researches about the microalgae, no study have focused on the potential mechanism of LDHs toxicity to *T. obliquus*.

The present work aimed to determine the toxicological impacts of ZnFe-SO_4_ LDH on *T. obliquus*, as well as to screen some biochemical and physiological responses of the algae to the existence of LDH in its environment. Toward this goal, the ZnFe-SO_4_ LDH was synthesized and characterized by SEM (Scanning electron microscopy), EDX (Energy dispersive X-ray) mapping, TEM (Transmission electron microscopy), XRD (X-ray diffraction) and FT-IR (Fourier transform infrared). Next, after the treatment of the algae with ZnFe-SO_4_ LDH, the toxicological effects of the contaminants on some algal physiological parameters, including growth, pigments content and enzymatic and non-enzymatic antioxidants, were determined.

## Results and discussion

### Characterization

**Figure 1:**
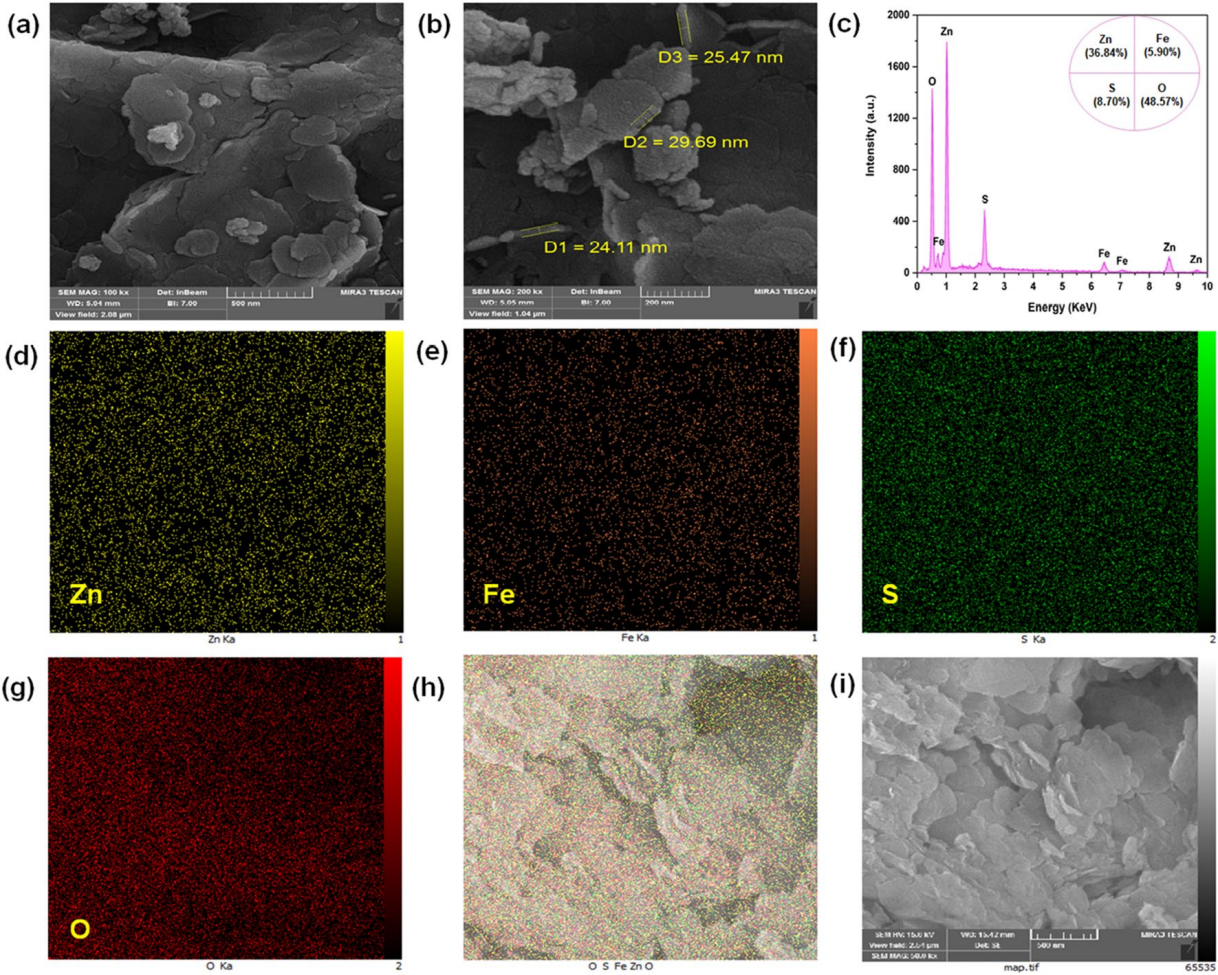
FESEM images of ZnFe-SO_4_ LDH with different magnifications (**a** and **b**), and (**c**–**i**) EDX-mapping analysis of ZnFe-SO_4_ LDH (The scale bars are 500, and 200 nm).

To examine the surface morphology and elemental composition of ZnFe-SO_4_ LDH, FESEM and EDX analyses were performed, respectively. The uniformness of the nanomaterials properties is crucial^19^. Figure 1a,b, displays the formation of nanosheets (average thickness of about 26 nm). The successful synthesis of ZnFe-SO_4_ LDH exhibited characteristic hexagonal plate-like LDH sheets and was predominately smooth. The results of ZnFe-SO_4_ LDH were in a close agreement with the corresponding literature data^20,21^. Furthermore, for ZnFe-SO_4_ LDH to check the possible impurity elements from the synthesis processes, EDX-map measurements were performed^22^. The results of the EDX-mapping microanalysis are indicated in Fig. 1c–i. The presence of Zn, Fe, S, and O in its spectra confirmed the successful synthesis of ZnFe-SO_4_ LDH without other impurities. Due to the ZnFe-SO_4_ LDH being synthesized with a ratio of 3:1, the presence of Zn, Fe, S, and O was 36.84%, 5.9%, 8.7%, and 48.57% in the LDH structure, respectively. Also, the elemental mapping images revealed that Zn, Fe, S, and O were homogeneously and uniformly distributed throughout ZnFe-SO_4_ LDH parallel with the amounts used in the synthesis.

**Figure 2:**
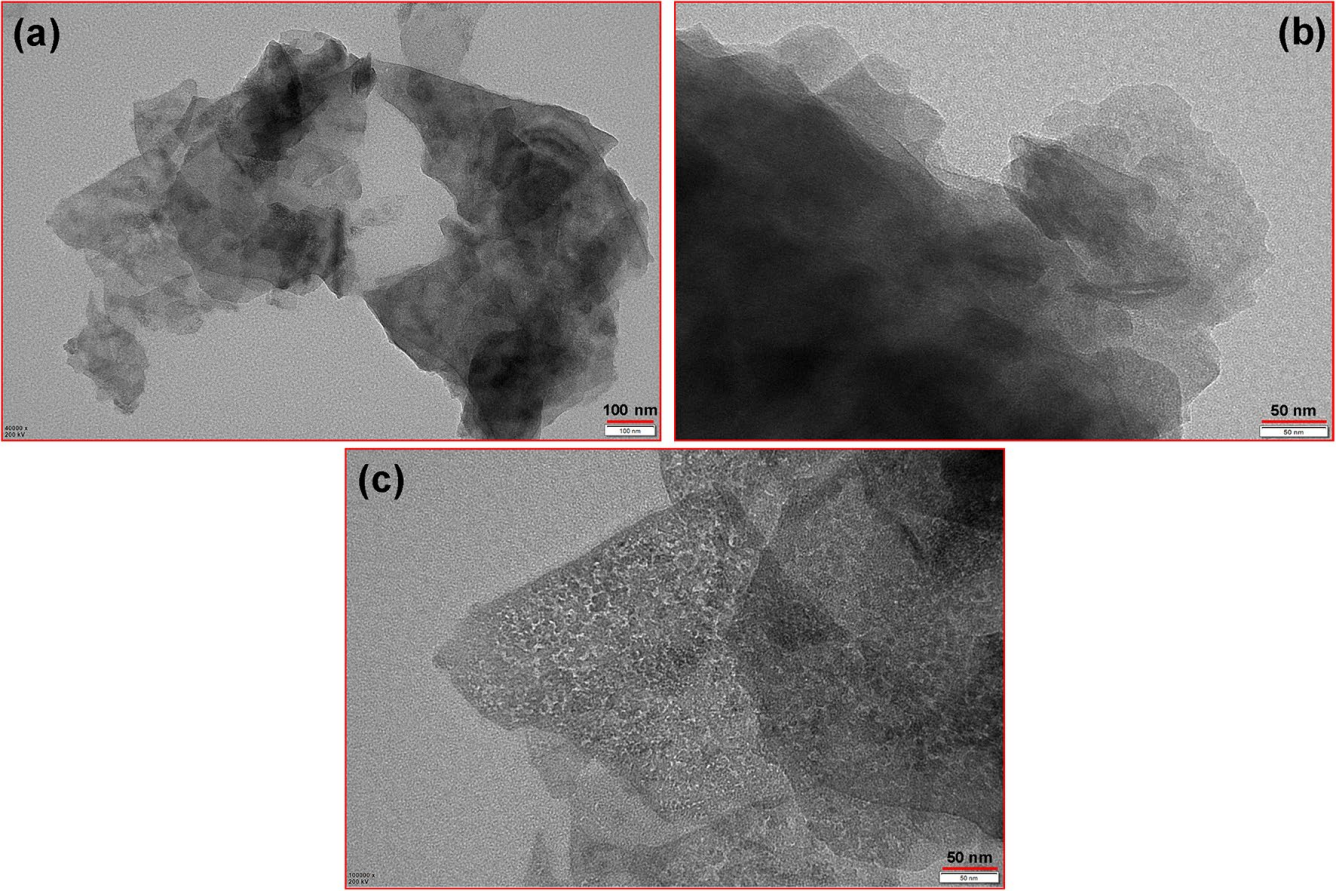
TEM images of ZnFe-SO_4_ LDH with different magnifications (The scale bars are 50, and 100 nm).

The morphology of the as-synthesized samples was measured by TEM images through different magnifications (Fig. 2). The TEM mode is well-suited for crystal orientation analysis and periodic structure analysis of nanomaterials. In addition, diffraction patterns obtained from TEM mode can provide valuable insights into the crystal structure of the sample. The TEM images showed that the prepared plate-like ZnFe LDH included crystallites which were approximately hexagonal. The structure indicated the layered essence of the LDH.

**Figure 3:**
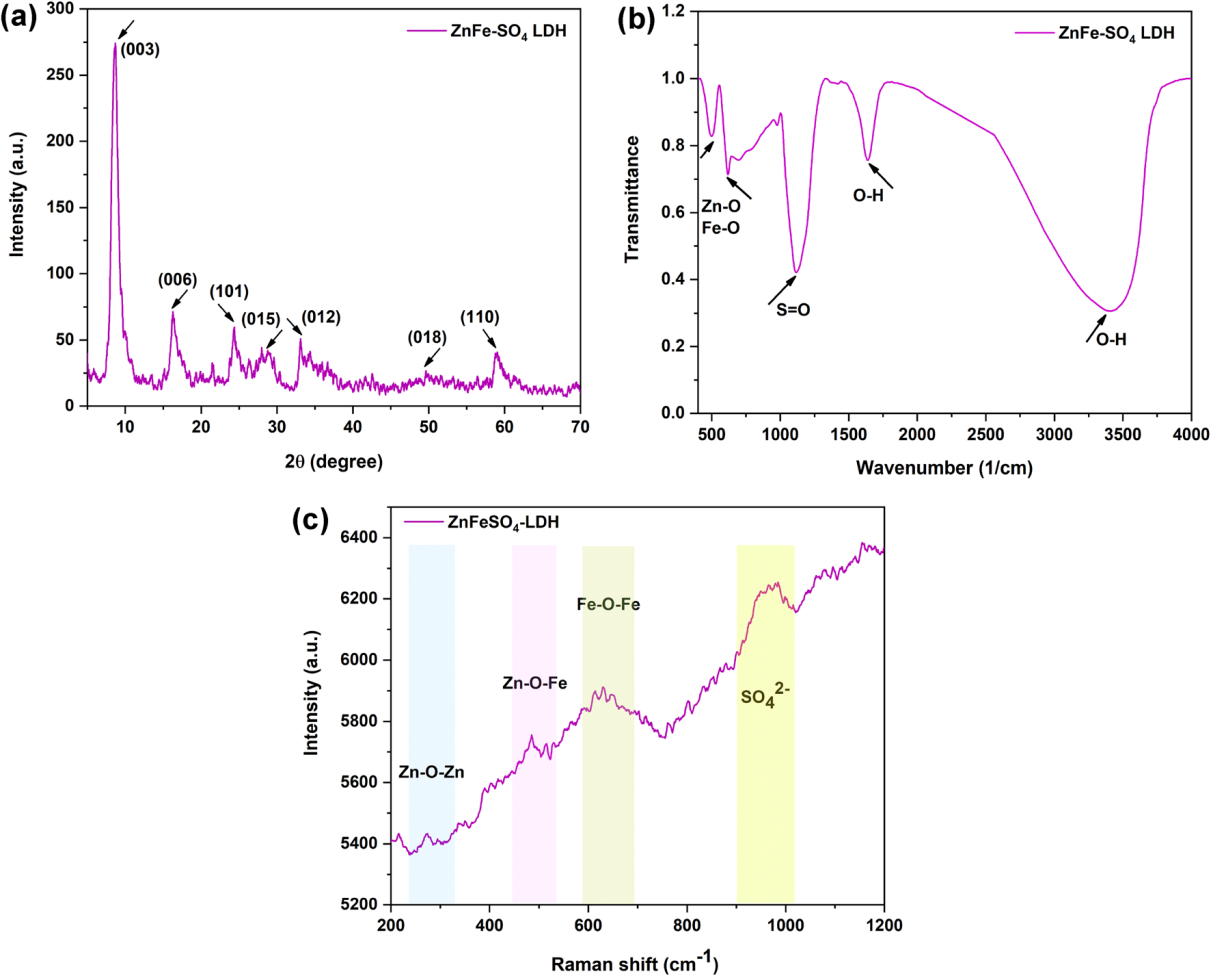
(**a**) XRD, (**b**) FT-IR, and (**c**) Raman spectra of ZnFe-SO_4_ LDH.

XRD analysis was then carried out to characterize the crystalline structure and phase change of ZnFe-SO_4_ LDH. For the ZnFe-SO_4_ LDH, the peaks located at 9.7°, 16.26°, 24.32°, 29.12°, 33.08°, 42.34°, 49.64°, and 59.1° corresponded to the reflections of the (003), (006), (101), (015), (012), (018), and (110) planes of LDH, which were typical of the characteristic pattern of hydrotalcite (Fig. 3a). The (003) and (006) planes indicated the incorporation of carbonate ions and H_2_O in LDH lattice. Similar to the obtained results, the ZnFe LDH showed successful characteristic reflections corresponding to the crystalline layered phase of the LDHs in other previous studies^20,23^.

The functional groups and the molecular structure of ZnFe-SO_4_ LDH were determined via FTIR analysis (Fig. 3b). The –OH stretch vibrations with hydrogen bonding and surface water molecules have been detected with the broad, strong bands within the range of 3448 1/cm. The interlayer water bending modes could be identified by the bands in the range of 1645 1/cm. The bands located at 1128 1/cm were related to sulfate ions. The peaks showed stretching and vibration modes of M–O and O–M–O in the hydrotalcite around 400–800 1/cm and 400–600 1/cm, respectively, where M denotes the cations of Zn^2+^ and Fe^3+^^24^.

Raman spectroscopy provides identification of crystalline phases present in materials from the number of observed bands and their wave numbers which are related to their space group and the bonds force constants, respectively. Usually, the Raman spectrum of a nanomaterial remains sufficiently similar to the corresponding single-crystal one to allow univocal identification. The interlayer anions of LDH structures are characterized by the weak band at 900–1000 cm^−1^ in the Raman spectrum (Fig. 3c). The lattice vibration modes of M–O and O–M–O, could be identified by the bands in the low-frequency area. The existence of the peak at about 600 cm^−1^ was related to the Fe–O–Fe stretching vibrations. Also, the peak at 300 cm^−1^ and 450 cm^−1^ could be assigned to Zn–O bonds and Zn–O–Fe bonds, respectively^25^.

### ZnFe-SO_4_ LDH effect on algal cell growth and pigments, content

**Figure 4:**
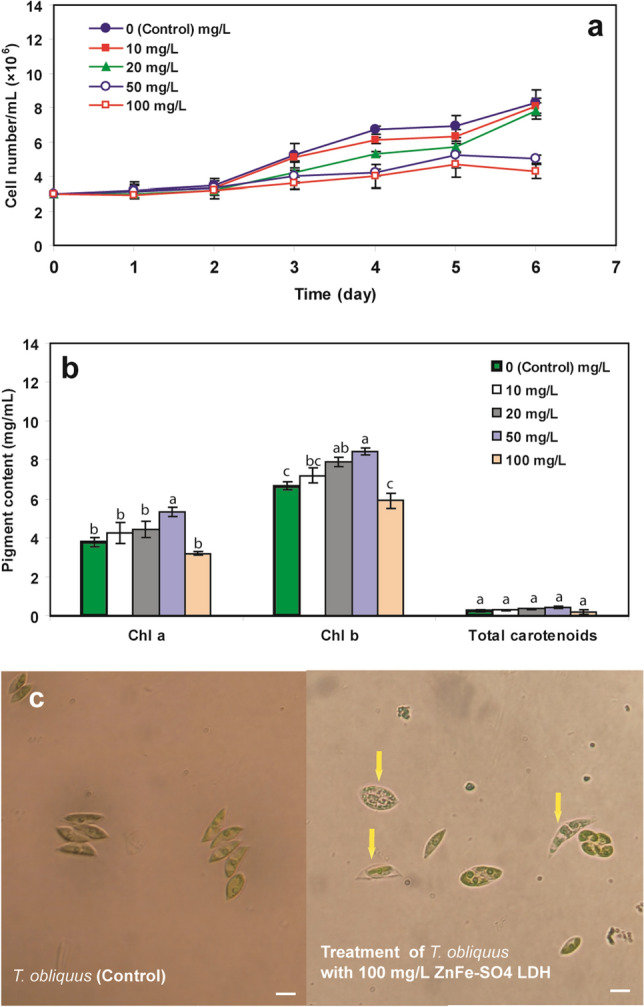
The effect of different concentration of ZnFe-SO_4_ LDH (mg/L) on (**a**) growth and (**b**) contents of chlorophyll a, b, a + b and total carotenoids of *T*. *obliquus* (Mean ± SD, n = 3, different letters in each column showed statistically significant difference (*p* < 0.05)) and (**c**) Light microscopic images of *T. obliquus* cells in control condition (left) and after treatment with 100 mg/L ZnFe-SO_4_ LDH (The scale bars are 10 μm; yellow arrows show the vacuolization of the cells).

Nanomaterials would inhibit algae's growth by affecting gene expression, metabolism, photosynthesis and nitrogen fixation. The inhibition of algae growth may vary with the type, concentration, exposure time of nanomaterials and testing organisms^9^. The growth profiles of the algal cells grown under treatment with 10 and 20 mg/L of ZnFe-SO_4_ LDH for six days were similar (Fig. 4a), and these concentrations seldom imposed a significant effect on the cell growth. Treatment of concentrations higher than 20 mg/L (50 and 100 mg/L ZnFe-SO_4_ LDH) reduced the final cell density to near 40% and 48.2%, respectively. In general, the higher exposure concentration of LDH would induce inhibition of the microalgae growth. The toxicity of some other nanomaterials, such as carbon nanotubes, Fe_2_O_3_ nanoparticles and MgO nanoparticles was also reported for various green algae. For instance, MgO nanoparticle was found to be toxic to *S. obliquus* and *Pseudokirchneriella subcapitata* even at low (0.8 mg/L) and high concentrations (100 mg/L), respectively^26,27^. Different sensitivity of algal species to various concentrations of nanomaterials was also observed for iron-based nanoparticles. The toxic effects of Fe_2_O_3_-NP on *Nanochloropsis* sp. and *Isochrysis* sp. appeared at low concentrations^28^; meanwhile, in *Chlorella vulgaris*, the high concentration of nanoparticles caused toxicity effects^29^. These various responses of algal species to different concentrations of the contaminants might be a species-specific behavior shared by virtually all members of a given species^27^.

After exposure of the algae cell to different concentrations of ZnFe-SO_4_ LDH, the content of photosynthetic pigments was determined. Treatment of the algal cells by 10, 20 and 50 mg/L of the pollutant led to the enhancement of Chl *a*, *b*, as well as total carotenoids contents, as compared to the control experiment (Fig. 4b). The increase of Chl *a* and *b* content was statistically significant only for 50 mg/L of the pollutant (*p* < 0.05). Treatment of 100 mg/L of the contaminant reduced Chl *a* and *b*, as compared to the control sample. Chlorophyll content is one of the important indicators confirming the tension situations in photosynthetic organisms and its depletion can lead to the reduction of the photosynthetic rate when followed by the decrement in the growth rate of the given organism^30^. In contrast to Chl changes, the total carotenoids were raised at all concentrations of ZnFe-SO_4_ LDH (Fig. 4b), but these increases were not statistically significant (*p* > 0.05). According to the literature, the carotenoid content was also enhanced in *C. vulgaris* cells after treatment with some nanoparticles such as ZnO and Fe_2_O_3_ during heavy metals stress^31,32^. Moreover, it was shown that despite Ni negatively affects on chlorophyll contents, it increases carotenoids content in *Dunaliella* sp^33^. An increase in the amount of some antioxidant compounds, including carotenoids, is one of the events that can occur following undesirable environmental conditions, such as the existence of pollutants in the environment^34^. Carotenoids protect the integrity of the cell membranes and increase their stability by inhibition of lipid peroxidation^35,36^.

Figure 4c also illustrates light microscopic images of the microalgae in control samples and after exposure with 100 mg/L ZnFe-SO_4_ LDH. The adsorption of nanomaterials to cell surface or their entrance to algal cells and destruction of the subcellular structures were reported as adverse effects of nanomaterials in algae^9^. In the present study, the treated cells showed varying degree of vacuolization and increased cavitations, as compared to the images of the control cells (Fig. 4c). However, the exact effects of the LDH on cell organelles is not clear and needs further investigation.

### Antioxidants assays

**Figure 5:**
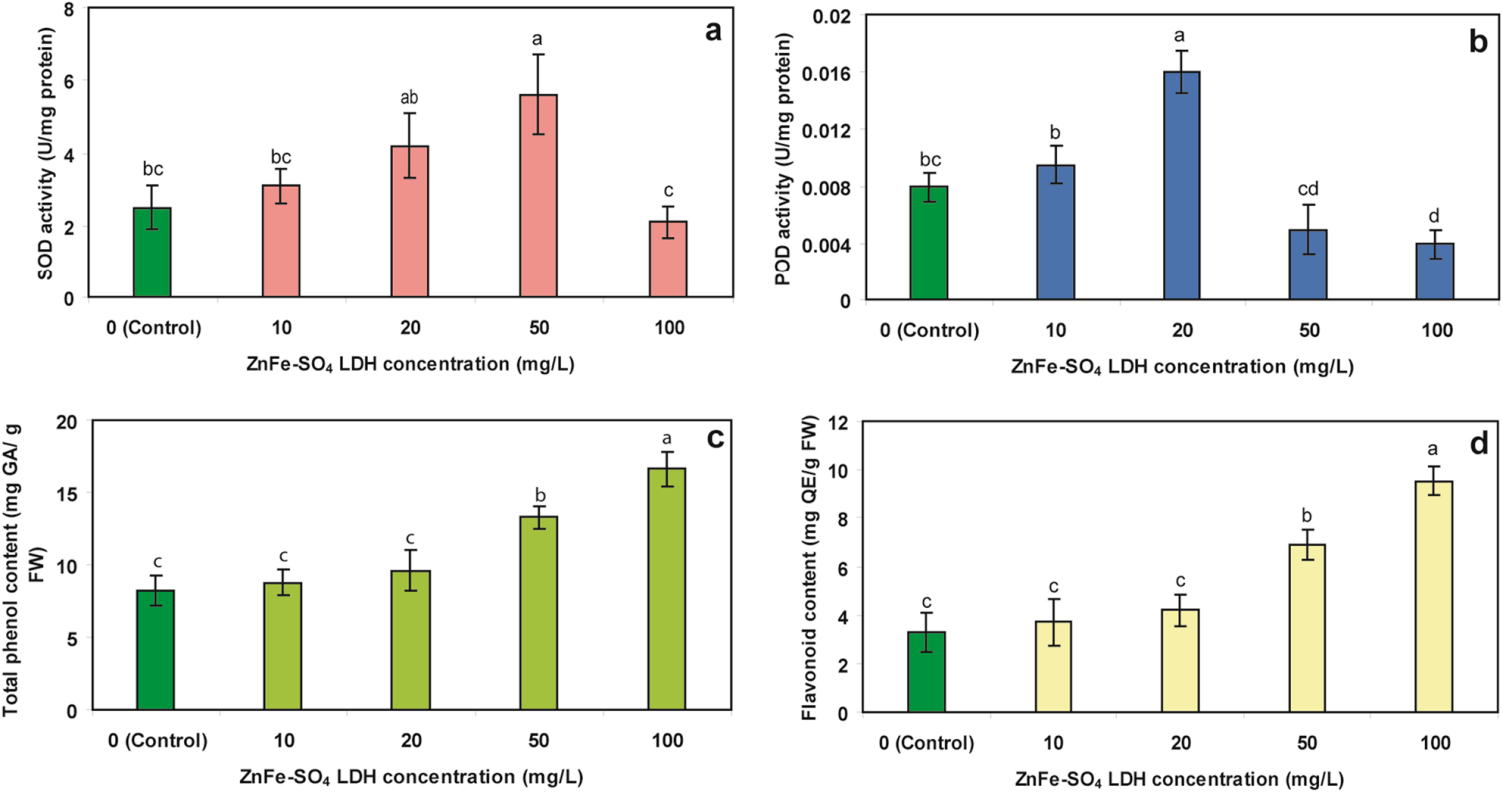
(**a**) SOD and (**b**) POD activities and the content of (**c**) total phenol and (**d**) flavonoids in control microalgae and the microalgae exposed to different concentration of ZnFe-SO_4_ LDH (Mean ± SD, n = 3, different letters in each part of the figure showed statistically significant difference (*p* < 0.05)).

**Figure 6:**
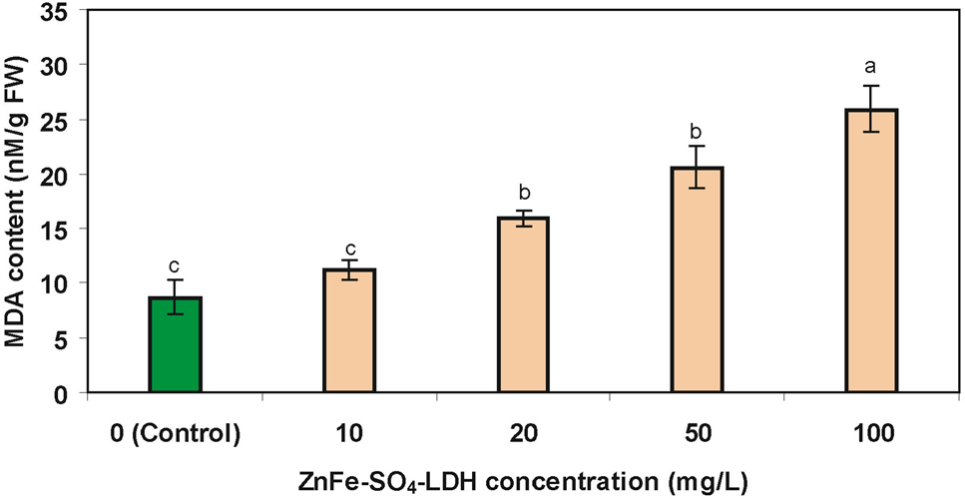
Contents of MDA (nM/ g FW) in control *T. obliquus* and the microalgae exposed to different concentrations of ZnFe-SO_4_ LDH [Mean ± SD, n = 3, different letters in each column showed statistically significant difference (*p* < 0.05)].

Figure 6 illustrates the response of SOD and POD enzymes of the algae cells following the contaminant exposure. Accordingly, SOD activity was significantly enhanced after treatment by 50 mg/L ZnFe-SO_4_ LDH, resulting in the 2.2-fold induction of the SOD activity, as compared to the corresponding control (Fig. 5a). Augmentation and induction of SOD activity were reported after treatments of different concentrations of nonomaterials such as carbon nanotubes, Fe_2_O_3_ nanoparticles and ZnO nanoparticles in *S. obliquus* and *C. vulgaris*^27,32^. The induction of SOD activity was also reported in different microalgae species in response to heavy metal stress. A concentration-dependent increase in SOD activity has been revealed with Cu, Pb and Cd in *C. vulgaris* and with Cu and Zn in *Pavlova viridis* and *S. vacuolatus*^37–39^. However, the enzyme activity was reduced significantly after six days of treatment with 100 mg/L ZnFe-SO_4_ LDH. In biological systems, SOD, as a front line of defense mechanisms, is involved in combatting reactive oxygen species (ROS) produced during different stress conditions^40^. Thus, an increase in SOD activity has been correlated with the mechanisms responsible for tolerating and coping the stress conditions^41^.

POD is one of the most important enzymes for ROS capture in cell of microalgae under heavy metal stress and works in close synchrony with SOD to prevent production of more ROS during different stresses^42^. Treatment of 10 and 20 mg/L of the LDH led to increase of POD activity, but contrary to the induction of the SOD activity after treatment with 50 mg/L ZnFe-SO_4_ LDH, the treatment of 50 mg/L along with 100 mg/L lowered the POD activity (Fig. 5b). Increased POD activity has been shown in different microalgae such as *C. vulgaris, S. obliquus* and *Dunaliella* sp. under exposures to Cu, Cd, Ni and Pb^15,33,37^. In contrast to such induction of antioxidant enzymes activities, their inhibition following the treatment of living organisms with high concentrations of contaminants has been confirmed in other studies^40^.

Due to the structural properties of phenolic compounds, such as aromatic rings in their chemical structures, they can act as radical scavengers^43^. According to Fig. 5c, some concentration-dependent increase was observed in the amounts of phenols and flavonoids. The phenol content in the microalgae was elevated up to 13.3 and 15.6 mg GA/g FW after treatment with 50 and 100 mg/L ZnFe-SO_4_ LDH, respectively (compared to its content in the control sample, 8.2 mg GA/g FW). Furthermore, a significant enhancement in the flavonoids content of the algae was observed after its treatment with 50 and 100 mg/L ZnFe-SO_4_ LDH (Fig. 5d). Although, unlike plants, there is not much information about the type and content of phenolic compounds in algae and about these compounds involvement in cellular responses to ROS generated during different stresses^44^. It was found that the phenolic profile of diatom *Phaeodactylum tricornutum* was dependent upon the addition of copper and iron to the culture media and the production of relevant amounts of phenolic compounds acted as protective mechanism against toxicity of Cu and Fe^45^. The accumulations of gentisic acid, (+) catechin and (−) epicatechin, the most prominent phenolic compounds, in *Dunaniella tertiolecta* have also been previously reported after the algae treatment with 790 nmol/L Cu^46^. In addition to the phenolic compounds, the augmentation in flavonoids content was reported after the treatment of *C. sorokiniana* and *S. acuminatus* by Zn^47^.

### ZnFe-SO_4_ LDH effect on the MDA content

One of the main destructive effects of ROS is removing hydrogen from unsaturated chain of fatty acids and causing their additional peroxidation at the cell membrane. During lipid peroxidation, different types of cytotoxic compounds such as MDA are produced. Therefore, MDA content is directly correlated with ROS accumulation and subsequent oxidative damage^9^. Treatment of microalgae with 20, 50 and 100 mg/L ZnFe-SO_4_ LDH led to a statistically significant rise of the MDA content up to 15.9, 20.6 and 25.9 nM/gFW, respectively (Fig. 6). The obtained result could be a sign of lipid peroxidation during the treatment of the algae with high concentrations of the pollutants. MDA has been extensively studied in different microalgae under various stress conditions and similar effects were detected after exposure of microalgae with different pollutants, such as other nanomaterials, heavy metals and antibiotics. For instance, MDA content was raised in *S. obliquus* and *Raphidocelis subcapitata* after exposure to high concentrations of carbon nanotubes and clarithromycin, respectively^27,48^. Moreover, exposure of *S. obliquus* and *Euglena gracilis* to Pb and Cr at their EC_50_ concentrations increased the MDA content respectively^15,49^.

## Methods

### LDH synthesis

ZnFe-SO_4_ LDH was synthesized via the co-precipitation method. FeSO_4_.7H_2_O (0.001 mol) and ZnSO_4_.7H_2_O (0.003 mol) were dissolved in 40 mL distilled water. The solution was titrated slowly with NaOH solution (2 mol/L) until the pH reached 8 at room temperature and under Ar atmosphere. After 24 h stirring, the precipitates were centrifuged and dried overnight in an oven at 60 °C^3^.

X-ray diffraction (XRD) (Tongda-TD-3700, China, Cu Kα radiation: λ = 1.5406 Å; 30 kV, 20 mA) was used to study the crystallographic characteristics of the samples. Fourier-transform infrared spectroscopy (FTIR) was also applied to measure the samples' functional groups within the 400–4000 cm^−1^ range via the KBr disk method (Tensor 27 spectrometer, Bruker, Germany). Field emission scanning electron microscopic (FESEM) micrographs and transmission electron microscopic (TEM) images of the samples were obtained using a Tescan Mira3 microscope (Czech Republic) and A Jeol JEM2011 microscope, respectively. EDX-mapping analysis was then performed by a Zeiss Sigma 300. Raman measurement was also performed (Raman, ANDOR, Umi DRON-A, Korea) with a laser (532.15 nm). The thickness of the synthesized ZnFe-SO_4_ LDH was determined by the Digimizer software (version 4.1.1.0).

### Algae cultivation and treatments

*T. obliquus* was obtained from the Artemia and Aquaculture Research Institute, Urmia University. As mentioned in the text, *T. obliquus* was obtained from the Artemia and Aquaculture Research Institute, Urmia University. Identification of the microalgae, was already done by Asalpishe et al.^50^. Briefly, using pairs of primers designed by Baldwin^51^, two segments of ITS (Internal transcribed spacer) (ITS1 and ITS 2) were amplified by PCR. For obtaining the precise sequence of PCR products, Sanger sequencing was applied. The sequence of the amplified region was submitted into NCBI with accession number OR393092. The microalgae was cultivated in 100 mL liquid mineral Bold's Basal Medium (BBM) using 250 mL flasks^52^. The flasks were at 22 ± 2 °C, illuminated with cool white fluorescent tubes at a continuous light intensity of 60 μE/m^2^/s (24 h photoperiod) and aerated with filtered air continuously. The microalgae were harvested every seven days. The flasks containing specific numbers of *T. obliquus* and culture medium were exposed to 10, 20, 50 and 100 mg/L of ZnFe-SO_4_ LDH, during the experiment.

### Measurement of the growth rate and pigments content

In order to perform the microalgae growth assay, pre-cultures of *T. obliquus* at the exponential phase were inoculated into the fresh culture medium containing different concentrations of ZnFe-SO_4_ LDH (initial cell density: 10^6^ cells/mL). The treatment lasted for seven days and the algal growth was monitored daily using a hemocytometer with Neubauer improved rulings (Boeca, Germany), after fixation of the microalgae cells with Lugol’s solution.

The 2 mL micro-algae culture was centrifuged and after decanting the supernatant, the residue sediment was mixed with acetone 100% and then incubated for 24 h without light. Finally, the contents of Chl *a*, *b*, as well as total carotenoid were determined spectrophotometrically using the equations described by Lichtenthaler^53^.

### Antioxidants assays

To evaluate the antioxidant responses, *T. obliquus* cells were treated with different concentrations of ZnFe-SO_4_ LDH suspensions in the nutrient solution. The harvested cells from 100 mL culture medium were homogenized in a buffer solution containing 0.2% polyvinylpyrrolidone (PVP) and centrifuged at 4 °C. The resulting supernatants were applied to assess the activities of antioxidant enzymes. Bradford assay was then applied for protein content measurement^54^.

The SOD activity was acquired by determining the inhibition of the photochemical reduction rate of nitroblue tetrazolium (NBT) through the algal extract. The reaction buffer contained 67 mmol/L potassium phosphate buffer solution (pH 7.8), 1.5 mmol/L NBT, 0.12 mmol/L riboflavin and a suitable aliquot of enzyme extract. The reaction mixture was illuminated for 15 min at light intensity of 5000 Lux. The absorbance was measured at 560 nm and the enzyme activity was indicated by U/mg protein^55^. To determine POD activity, a reaction mixture was used composed of guaiacol (ɛ: 26.6 mM^−1^ cm^−1^), H_2_O_2,_ citrate–phosphate-borate buffer and a suitable amount of enzyme extract^56^. The increase in absorbance at 470 nm during polymerization of guaiacol to tetraguaiacol was recorded for 3 min.

The content of the total phenols was calculated by a spectrometric method described by Singleton^57^. A proper amount of the algae extract was mixed with 600 μL of distilled water; then, 50 μL of the Folin-Ciocalteu’s reagent was added to the mixture. After 7 min, 150 μL Na_2_CO_3_ (20%) was added and the total volume of the mixture was 1 mL by distilled water. After 2-h incubation of the samples at the ambient temperature, the absorbance was determined at 760 nm. Their contents were expressed as mg of gallic acid equivalent (GAE) per g of the algae fresh weight (FW). To determine the flavonoids content, the aluminum chloride colorimetric method^58^ by quercetin as a reference compound was used. To put it briefly, 600 μL of the algae methanolic extract (1 mg/mL) was mixed with 2% AlCl_3_ methanol solution. After the 60 min incubation at room temperature, the absorbance of the mixture was determined at 420 nm and expressed as mg quercetin equivalent (QE) g^−1^ FW.

### Malondialdehyde (MDA) content

To determine malondialdehyde (MDA) accumulation, 0.01 g of fresh algae samples was ground in 1 mL of 0.1% (w/v) trichloroacetic acid (TCA), at 4 °C; then, a suitable amount of 0.5% (w/v) thiobarbituric acid (TBA) in 20% (w/v) TCA was added and heated at 95 °C for 30 min. The reaction mixture was refrigerated rapidly and centrifuged at 10,000*g* for 10 min (4 °C). The supernatant absorbance was determined at 532 nm and 600 nm and MDA content was expressed as nmol MDA g^−1^ FW (ɛ = 155 mM^−1^ cm^−1^) according to the method developed by Heath and Packer^59^.

### Statistical analysis

One-way ANOVA followed by Tukey–Kramer, multiple comparisons test, was used for statistical data analysis (data with three replicates; using GraphPad Software, Inc. USA). The results were described as mean ± standard deviation (SD).

## Conclusion

The toxicity of ZnFe-SO_4_ LDH to a typical microalgae *T. obliquus* was determined. The growth inhibition and reduction of the chlorophyll contents were found when the microalgae was exposed to high concentration of the LDH. The LDH could also cause lipid peroxidation and induce the antioxidant defense system of *T. obliquus* to protect the microalgae from the undesirable toxic effects of the pollutant. The contents of total phenols and flavonoids were increased in response to treatment of high concentration of the LDH and possibly due to the induced ROS production during the high dose exposure to the pollutant. Furthermore, the activities of SOD and POD enzymes were inhibited after the treatment of *T. obliquus* with 100 mg/L of ZnFe-SO_4_ LDH. The results of the present study will be helpful for the risk assessments of LDHs and provide a new insight into the toxicological effects of LDH in algae.

## Data Availability

The dataset used in the current study is available upon reasonable request through the following emails contact: s.torbati@urmia.ac.ir and a_khataee@tabrizu.ac.ir.
